# Soil–Climatic Drivers of Anatomical and Metabolic Plasticity in *Rheum tataricum* L.f. Across Arid Landscapes of Kazakhstan

**DOI:** 10.3390/plants15071025

**Published:** 2026-03-26

**Authors:** Nina Terletskaya, Aigerim Mamirova, Yuliya Litvinenko, Meruyert Kurmanbayeva, Svetlana Polyakova, Nadezhda Gemejiyeva, Timur Kulmanov, Vitaliy Salnikov, Aizhan Mussayeva

**Affiliations:** 1 Faculty of Biology and Biotechnology, Al-Farabi Kazakh National University, Al-Farabi 71/19, Almaty 050040, Kazakhstan; aigerim.mamirova@mail.com (A.M.); meruyert.kurmanbayeva@kaznu.kz (M.K.); 2 Institute of Genetic and Physiology, Al-Farabi 93, Almaty 050040, Kazakhstan; rumex1978@gmail.com (Y.L.); aimus_@mail.ru (A.M.); 3 Faculty of Chemistry and Chemical Technology, Al-Farabi Kazakh National University, Al-Farabi 71/19, Almaty 050040, Kazakhstan; 4 Faculty of Geography and Environmental Sciences, Al-Farabi Kazakh National University, Al-Farabi 71/19, Almaty 050040, Kazakhstan; polse2468@gmail.com (S.P.); vitali.salnikov@kaznu.edu.kz (V.S.); 5 Institute of Botany and Phytointroduction, Almaty 050040, Kazakhstan; ngemed58@mail.ru

**Keywords:** *Rheum tataricum* L.f., ecopopulations, climate sensitivity, anatomical traits, metabolomic plasticity

## Abstract

Climate change and progressive aridification represent a substantial threat to the sustainability of wild medicinal plant resources in Central Asia. *Rheum tataricum* L.f. (*R. tataricum*), a mesoxerophytic species with high pharmacological potential and a restricted distribution range, was selected as a model for investigating adaptive responses to combined climatic and edaphic stress. Relationships among climatic parameters, soil agrochemical characteristics, anatomical and morphological traits, and the metabolomic profile of roots and rhizomes were analysed across six ecopopulations distributed along latitudinal and altitudinal gradients in southern and western Kazakhstan. To quantify population-level vulnerability to climatic stress, a Climate Sensitivity Index (CSI) was calculated. All investigated ecopopulations exhibited high climate sensitivity (CSI = 0.30–0.40), indicating persistent climatic stress. Significant altitudinal dependence was detected for such anatomical traits, as primary cortex thickness, as well as for the accumulation of tannins, anthraquinones, and flavonoids. The metabolomic profile was strongly associated with seasonal precipitation, temperature, relative air humidity, soil agrochemical properties, and root elemental composition. These findings demonstrate pronounced anatomical and metabolomic plasticity in *R. tataricum*, which appears to function as a key adaptive mechanism in arid ecosystems. The results provide a scientific basis for sustainable bioprospecting, conservation of natural populations, and targeted cultivation aimed at obtaining specific metabolomic profiles.

## 1. Introduction

Global plant biodiversity is increasingly exposed to a complex array of abiotic and anthropogenic stressors, including soil salinisation and degradation, environmental pollution, urbanisation, rising air temperatures, and ongoing climate change [1]. These processes exert direct effects on the stability of natural ecosystems and on the availability of plant resources, including medicinal species, whose chemical diversity has been shaped through long-term selection under natural environmental stress. Within this framework, particular attention should be directed towards species that combine high pharmacological value with pronounced ecological specialisation, as such taxa are typically the most sensitive to alterations in climatic and edaphic conditions.

Among the numerous plant species recognised for their medicinal properties since early civilisations, rhubarb (*Rheum* spp.) remains one of the few that continues to be widely utilised in both traditional and official herbal medicine. The earliest documented references to rhubarb occur in ancient Chinese texts dating to approximately 2700 BC. Owing to its diverse therapeutic applications, this pharmacologically active genus rapidly gained prominence and became one of the most valuable commodities traded along the Silk Road [2,3].

Numerous studies have demonstrated that species of the genus *Rheum* exhibit a broad spectrum of pharmacological activities, including cardioprotective, anticancer, hepatoprotective, nephroprotective, pulmonary protective, antidiabetic, anti-colitic, and antibacterial effects. Therapeutic preparations derived from rhubarb have been shown to improve clinical outcomes through modulation of microbial dysbiosis, restoration of aberrant non-coding RNA expression, and regulation of metabolic disorders, thereby offering potential targets for the treatment of a wide range of diseases [4,5].

Considerable research attention has been directed towards the roots and rhizomes of rhubarb, whose medicinal use was documented as early as 270 BC in the ancient Chinese pharmacopeia Shen Nong Ben Cao Jing [6]. Approximately 200 compounds have been isolated from the roots and rhizomes of different Rheum species, including anthraquinones, anthrones, stilbenes, flavonoids, acyl glucosides, tannins, and pyrones [7,8].

Modern investigations have elucidated the chemical composition of the roots and rhizomes of various rhubarb species [9], characterised the pharmacological activities of their bioactive constituents [10,11], and explored underlying mechanisms of action [8]. However, these studies have largely addressed isolated aspects of individual species, and the extent of investigation has varied considerably among taxa. In most cases, emphasis has been placed either on specific pharmacological effects or on phytochemical profiles of roots and rhizomes, without adequate consideration of the environmental context in which these traits are formed [7,8]. Yet phenotypic and metabolic plasticity are particularly critical for the underground organs of perennial plants, as roots and rhizomes function both as reservoirs of biologically active compounds and as principal structures conferring tolerance to abiotic stress [12,13].

The genus *Rheum* L. (family *Polygonaceae*) comprises approximately 60 species [4]. Among them, Tatarian rhubarb (*Rheum tataricum* L.f.) is characterised by a restricted distribution range, occurring predominantly in the dry steppes and deserts of Central Asia, including the Astrakhan region of Russia, central, southern and western Kazakhstan, western China (Xinjiang), and Afghanistan. This species belongs to a group of taxa highly adapted to semi-desert and desert environments, in which both morphological modifications (e.g., enlarged leaf area, reduced and deformed stems, and deep root systems) and biochemical traits contribute to plant survival under arid conditions [14]. In Kazakhstan, *Rheum tataricum* possesses considerable resource potential and represents the most widespread species of the genus *Rheum* L. [15].

The roots and rhizomes of *R. tataricum* contain carbohydrates, organic acids, phenols, catechins, tannins, anthraquinones, and higher aliphatic hydrocarbons [16]. In addition to its recognised medicinal applications, the species is of considerable importance as a forage plant. During early spring, when forage resources are limited, the succulent leaves serve as a valuable feed source for camels, sheep, goats, and horses [17,18]. Despite its recognised resistance to drought, salinity, and nutrient deficiency, and its acceptance in official medicine, *R. tataricum* remains one of the least studied wild rhubarb species [14,19].

Wild plant species such as *R. tataricum* have evolved over millions of years under natural selection in heterogeneous landscapes, where microclimatic gradients, fragmented resource availability, and variable pressure from herbivores and pathogens have necessitated the development of flexible, context-dependent chemical phenotypes. Under the unprotected conditions of natural populations, in which abiotic and biotic stresses prevail, biologically active compounds and secondary metabolites primarily function as a complex defensive system. An improved understanding of local plant adaptation provides a valuable framework not only for elucidating the evolutionary dynamics of phytochemical profiles but also for guiding their targeted modification during domestication and introduction. Populations of the same species distributed along altitudinal, latitudinal, or edaphic gradients frequently exhibit pronounced differences in phytochemical composition and concentrations of bioactive compounds, reflecting distinct selective regimes operating within specific microhabitats [20,21,22,23,24].

Despite the substantial body of knowledge concerning the phytochemistry and pharmacological properties of the genus *Rheum*, a significant gap persists due to the scarcity of integrative investigations that simultaneously consider climatic variables, soil characteristics, anatomical and morphological traits, and the metabolomic profiles of wild *R. tataricum* ecopopulations. A comprehensive understanding of how environmental factors induce or suppress the synthesis of specific combinations of bioactive compounds remains limited. Such knowledge is essential for sustainable bioprospecting aimed at identifying optimal populations for medicinal and forage use, conserving in situ chemical diversity, and developing cultivation systems capable of replicating key environmental signals to ensure the production of high-quality phytochemicals under laboratory and industrial conditions.

Accordingly, the present study aimed to elucidate relationships among climatic conditions across different *R. tataricum* habitats in Kazakhstan, soil agrochemical properties, anatomical and morphological characteristics of roots and rhizomes, and metabolomic phenotypes of distinct ecopopulations. For the first time, a quantitative assessment of climate vulnerability in *R. tataricum* populations was performed using the Climate Sensitivity Index (CSI), and comprehensive correlation analyses were conducted to associate variability in bioactive compounds with anatomical traits and environmental parameters.

The findings advance current understanding of adaptive mechanisms in *R. tataricum* under arid conditions and provide a scientific foundation for evidence-based bioprospecting, sustainable management of natural populations, and the development of cultivation strategies targeting specific phytochemical profiles in the context of ongoing climate change.

## 2. Results

### 2.1. Climate Sensitivity of Rheum tataricum Ecopopulations

Ecopopulations of *R. tataricum* in southern Kazakhstan were characterised by a marked decline in biomass yield and population density, accompanied by a shortened flowering period. The calculated Climate Sensitivity Index (CSI) values ranged from 0.314 to 0.400 across the assessed parameters (Table 1).

These characteristics indicate the presence of sustained climatic stress, which adversely affects both physiological processes and reproductive performance in plants.

### 2.2. Anatomical Structure of Rheum tataricum Rhizomes

**Figure 1:**
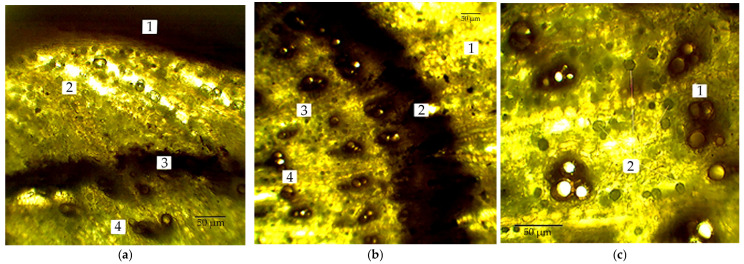
Anatomical section of *R. tataricum* rhizome. Notes: (**a**) 1—periderm; 2—primary cortex; 3—cambium; 4—section of central cylinder; (**b**) 1—primary cortex with inclusions, 2—cambium, 3—secondary phloem, 4—secondary xylem; (**c**) 1—secondary xylem vessels, 2—idioblasts in the central cylinder.

The anatomical structure of the *R. tataricum* rhizome at ×70 magnification is presented in Figure 1.

At the periphery of the transverse section, rows of darkly stained peridermal cells were observed. The periderm consisted of three distinct layers: phellem, phellogen, and phelloderm. Adjacent to the periderm, the primary cortex formed several concentric layers of parenchymatous cells containing numerous idioblast inclusions, which were abundant in all examined samples. The parenchyma cells of the primary cortex were rounded or rectangular, small to medium in size, with slightly thickened cell walls and lacking intercellular spaces.

The cambial zone was located at the boundary between the primary cortex and the central cylinder. Secondary phloem comprised axial and radial parenchyma arranged in a continuous concentric layer surrounding the cambium, with sieve elements organised into compact strands. The cambium was well defined and composed of densely packed cells. Secondary xylem consisted of radial chains of narrow, medium, and wide vessels converging towards the centre of the central cylinder; vessels were typically grouped in clusters of three to five. The central cylinder contained large pith parenchyma cells interspersed with alternating small and large idioblasts. Cross-sections of rhizomes from all populations exhibited a similar structural organisation. Numerous inclusions containing anthracene derivatives were detected throughout the parenchymatous tissue.

Morphometric differences among ecopopulations were associated primarily with periderm thickness, primary cortex thickness, and xylem vessel area (Table 2). Primary cortex thickness increased with increasing altitude, showing a 1.04–1.26-fold enhancement. However, above 450 m above sea level (a.s.l.), this parameter stabilised within the range of 26.6–26.8 µm. In contrast, periderm thickness and xylem vessel area did not demonstrate a stable, reliable altitude dependence, varying within 2.60–4.78 µm and 0.36–0.56 × 10^−3^ mm^2^, respectively, but maintained an increasing trend for each trait. The highest values for both traits were recorded in the Khantau population (558 m a.s.l.).

### 2.3. Metabolomic Profile of Rheum tataricum Ecopopulations

**Figure 2:**
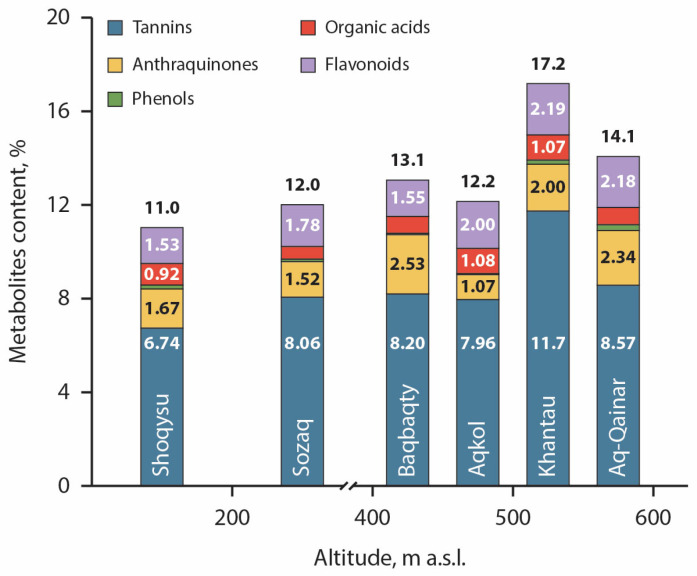
BACs content in the roots and rhizomes of *R. tataricum* from different collection sites.

For comparative analysis, the metabolomic profiles of the principal groups of biologically active compounds in *R. tataricum* were quantified (Figure 2).

The metabolomic profiles of roots and rhizomes varied significantly among ecopopulations and were strongly associated with altitude, as reflected in differences in the contents of tannins, anthraquinones (AQs), phenols, organic acids (OAs), and flavonoids. Among the analysed metabolites, three compound classes demonstrated a consistent increase with increasing elevation. Tannin content increased by 1.20–1.27-fold from 200 m a.s.l. and reached a maximum in the Khantau population (558 m a.s.l.), where a 1.74-fold increase was recorded. Anthraquinone content increased from 400 m a.s.l. by 1.20–1.51-fold, with the exception of the Aqkol population (441 m a.s.l.). Flavonoid accumulation increased by 1.31–1.43-fold at elevations above 450 m a.s.l.

### 2.4. Macro- & Micronutrient Accumulation in Rheum tataricum Rhizomes

The capacity of *R. tataricum* to accumulate essential for plant development elements (EEs) and potentially toxic elements (PTEs) was evaluated in relation to growth locality (Table 3).

Sodium (Na) and magnesium (Mg) accumulation ranged between 2.10–4.00 and 3.20–4.70 g kg^−1^, respectively, with no consistent altitudinal pattern observed. Similarly, potassium (K) and calcium (Ca) contents in the roots and rhizomes of *R. tataricum* varied within 7.40–9.40 and 30.2–49.0 g kg^−1^, respectively. Deviations from these ranges were recorded in the Aqkol and Aq-Qainar populations, which exhibited elevated K concentrations (20.1 ± 1.21 and 11.5 ± 1.27 g kg^−1^, respectively) and reduced Ca levels (18.4 ± 1.29 and 16.5 ± 1.63 g kg^−1^, respectively).

A comparable pattern was observed for manganese (Mn), with the Baqbaqty and Khantau populations showing lower concentrations (12.8 ± 2.30 and 18.2 ± 1.09 mg kg^−1^, respectively) compared with the typical range of 22.6–32.4 mg kg^−1^.

Regarding iron (Fe), higher accumulation levels (164–201 mg kg^−1^) were detected in the Shoqysu, Sozaq, and Aq-Qainar populations, whereas the remaining populations did not form a distinct cluster. Nickel (Ni), cadmium (Cd), and lead (Pb) concentrations in roots and rhizomes generally remained within the ranges of 3.00–3.70, 0.60–0.90, and 6.40–8.20 mg kg^−1^, respectively. However, the Baqbaqty population exhibited elevated Ni (9.40 ± 1.13 mg kg^−1^) and Pb (12.0 ± 0.84 mg kg^−1^) concentrations exceeding the typical range. In contrast, the Khantau population showed lower Cd (0.40 ± 0.04 mg kg^−1^) and Pb (3.90 ± 0.66 mg kg^−1^) contents. The Shoqysu population also demonstrated increased Ni accumulation (5.20 ± 0.31 mg kg^−1^).

Notably, zinc (Zn) was the only element exhibiting a clear altitudinal trend. At elevations above 500 m a.s.l., Zn concentrations increased markedly from the general range of 5.40–12.0 mg kg^−1^ to 71.5 ± 7.13 mg kg^−1^ in the Khantau population and 55.9 ± 5.03 mg kg^−1^ in the Aq-Qainar population.

### 2.5. Correlation Analysis of Rheum tataricum Parameters

#### 2.5.1. Correlation Between Altitude, Anatomical Traits, and Metabolomic Profile

**Figure 3:**
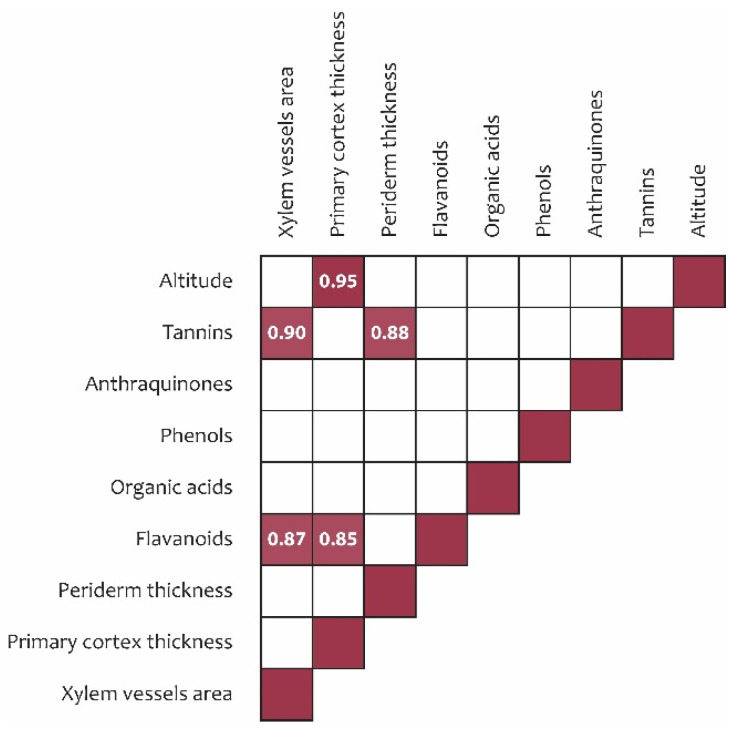
Pearson correlation between altitude, metabolomic profile, and anatomical traits. Note: only significant correlation coefficients are shown (*p* < 0.05).

Based on Pearson correlation analysis, primary cortex thickness was identified as the anatomical parameter most responsive to growth altitude, exhibiting a significant positive correlation (*r* = 0.95) and increasing with elevation (Figure 3).

Flavonoid content showed a strong positive correlation with primary cortex thickness (*r* = 0.85). In addition, tannin and flavonoid contents were positively correlated with xylem vessel area (*r* = 0.87 and *r* = 0.90, respectively), and tannin content was positively associated with periderm thickness (*r* = 0.88) (Figure 3).

#### 2.5.2. Correlation Between *R. tataricum* Parameters and Climatic Conditions

**Figure 4:**
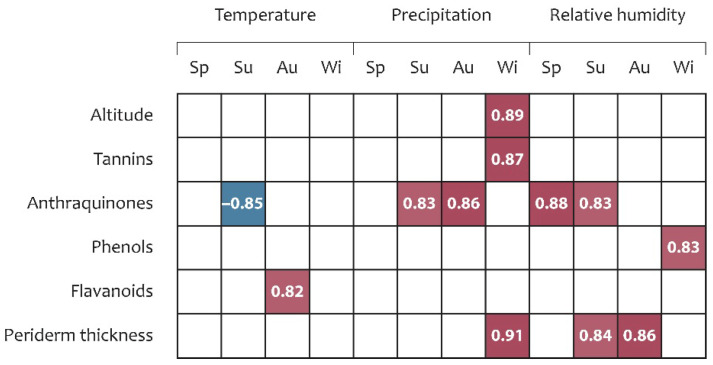
Pearson correlation between climatic parameters, metabolomic profile, and anatomical traits. Notes: only significant correlation coefficients are shown (*p* < 0.05); Sp—spring; Su—summer; Au—autumn; Wi—winter; in red—positive correlations; in blue—negative correlations.

Statistical analysis further indicated that the metabolomic profile was influenced by seasonal climatic variation in the growth localities (Figure 4). AQ content was the most responsive parameter, exhibiting positive correlations with summer (*r* = 0.83) and autumn (*r* = 0.86) precipitation, and with relative humidity in spring (*r* = 0.88) and summer (*r* = 0.83). A significant negative correlation was observed between AQ content and summer temperature (*r* = −0.85). Tannin, phenol, and flavonoid contents were positively correlated with winter precipitation (*r* = 0.87), winter relative humidity (*r* = 0.83), and autumn temperature (*r* = 0.82), respectively. Among anatomical traits, periderm thickness was positively correlated with winter precipitation (*r* = 0.91) and with relative humidity in spring and summer (*r* = 0.84 and *r* = 0.86, respectively; *p* < 0.05). In contrast, OA content, primary cortex thickness, and xylem vessel area did not demonstrate significant correlations with the climatic parameters examined (Figure 4).

#### 2.5.3. Correlation Between *R. tataricum* Parameters and Soil Agrochemical Profile

**Figure 5:**
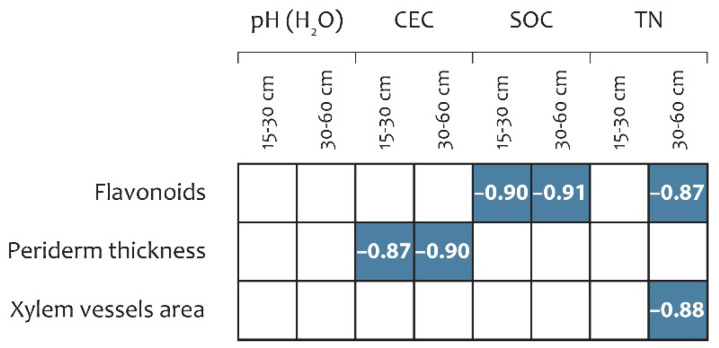
Pearson correlation between soil agrochemical parameters, metabolomic profile, and anatomical traits. Notes: only significant correlation coefficients are shown (*p* < 0.05); CEC—cation exchange capacity; SOC—soil organic carbon; TN—total nitrogen.

Pearson correlation analysis was performed to examine relationships among metabolic compounds (tannins, AQs, phenols, OAs, and flavonoids), anatomical traits (periderm thickness, primary cortex thickness, and xylem vessel area), and agrochemical characteristics of topsoil (15–30 cm) and subsoil (30–60 cm) sourced from predictive model SoilGrids, including soil pH, cation exchange capacity (CEC), soil organic carbon (SOC), and total nitrogen (TN). The analysis indicated that soil agrochemical factors were associated primarily with flavonoid content, periderm thickness, and xylem vessel area in *R. tataricum* (Figure 5).

All detected relationships were negative. Flavonoid content exhibited strong negative correlations with SOC in both soil horizons (*r* = −0.90 and −0.91) and with TN in the 30–60 cm horizon (*r* = −0.87). Likewise, xylem vessel area was negatively correlated with TN content in the subsoil (*r* = −0.88), whereas periderm thickness showed negative correlations with CEC in both soil horizons (*r* = −0.87 and −0.90).

#### 2.5.4. Correlation Between *R. tataricum* Parameters and Elements Accumulation Potential

**Figure 6:**
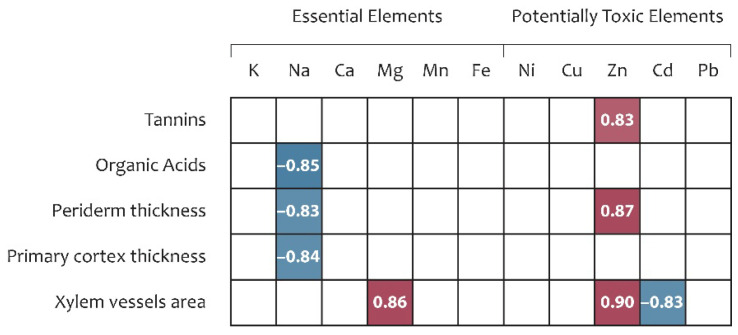
Pearson correlation between elements accumulation, metabolomic profile, and anatomical traits. Notes: only significant correlation coefficients are shown (*p* < 0.05); in red—positive correlations; in blue—negative correlations.

All assessed anatomical traits of *R. tataricum* demonstrated significant associations with elements essential for plant growth, as well as with PTEs (Figure 6). Periderm thickness (*r* = 0.87) and xylem vessel area (*r* = 0.90) were positively correlated with Zn accumulation in roots. In contrast, Na accumulation showed negative correlations with periderm thickness (*r* = −0.83) and primary cortex thickness (*r* = −0.84). Furthermore, xylem vessel area exhibited a positive correlation with Mg accumulation (*r* = 0.86) and a negative correlation with Cd accumulation (*r* = −0.83).

Among the analysed metabolites, only tannin and OA contents were associated with EE and PTE accumulation. Tannin content was positively correlated with Zn accumulation in roots (*r* = 0.83), whereas Na accumulation was negatively correlated with organic acid content (*r* = −0.85) (Figure 6).

## 3. Discussion

In the context of ongoing climate change, urbanisation, desertification, and environmental pollution, a comprehensive understanding of the diversity and spatial distribution of species with economic and medicinal significance has become increasingly critical. This issue is particularly relevant for Kazakhstan, where *Rheum* species are characterised by limited adaptive capacity and a narrow ecological amplitude [25].

### 3.1. Climate Sensitivity and Ecological Specificity of Rheum tataricum

A principal challenge in contemporary ecological research concerns the dynamic and complex environmental conditions of habitats occupied by species such as *R. tataricum*, where plants are exposed to multiple and interacting abiotic stressors. Under such circumstances, the evaluation of isolated adaptive mechanisms is insufficient to explain resistance to combined stresses [26].

*R. tataricum* is a mesoxerophytic species ecologically restricted to arid and semi-arid regions of Central Asia. The optimal temperature range for vegetative growth has been reported to be 0–25 °C, with a minimum annual precipitation requirement of approximately 130 mm. The establishment and persistence of its ecopopulations are closely associated with moisture deficit, and the generative phase represents the most critical stage of development. The CSI values obtained in the present study (0.314–0.400) indicate a high level of vulnerability to climatic stress, manifested in disturbances of physiological functioning and reproductive processes. Nevertheless, despite sustained climatic stress, *R. tataricum* maintains commercially viable populations within the studied localities. The underlying mechanisms responsible for this resilience warrant further consideration.

### 3.2. Anatomical Adaptations as the Basis for the Stability of Underground Organs

The persistence of wild plant species in heterogeneous landscapes is determined by their capacity for local adaptation. Evidence from the literature, together with the present findings, indicates that populations of a single species distributed along altitudinal, latitudinal, and edaphic gradients frequently exhibit pronounced differences in morphophysiological traits and metabolomic profiles, reflecting distinct selective pressures operating within specific microhabitats [12,13,21,24,27].

Parallel evolution of morphological traits within the genus *Rheum* has been proposed, with adaptation to contrasting environmental conditions driving trait diversification [28]. In the present study, periderm thickness, primary cortex thickness, and xylem vessel area were identified as informative anatomical traits that varied among ecopopulations.

A tendency towards increased periderm thickness with elevation was observed; however, this relationship was not statistically significant, possibly due to the influence of the outlier Aq-Qainar ecopopulation. This pattern may reflect enhanced rhizome resistance to desiccation, temperature fluctuations, and pathogen invasion. Meanwhile, greater primary cortex thickness observed at higher elevations (*r* = 0.85) was expected to improve water and mineral storage, as well as radial transport capacity. With respect to the response of xylem vessel area to increasing elevation, no significant trends were identified. Nevertheless, the enlargement observed in the Khantau and Aq-Qainar ecopopulations may facilitate more efficient upward water transport under conditions of periodic water deficit, although this response is likely associated with factors other than elevation.

### 3.3. Metabolomic Plasticity and the “Chemical Altitude Effect”

Intraspecific variability, defined as the diversity of trait values among individuals or populations within a single species, constitutes the primary substrate upon which natural selection acts and is particularly pronounced for specialised metabolites mediating interactions with climatic and edaphic factors [22]. Previous studies have demonstrated that even geographically proximate populations may differ markedly in their metabolomic profiles, underscoring the importance of subtle environmental and genetic influences [20,29].

Wild plants distributed along altitudinal gradients frequently exhibit the so-called “chemical altitude effect,” manifested as shifts in the content and composition of secondary metabolites and alterations in physiological stoichiometry [30,31,32]. Systematic altitudinal changes have been documented for anthraquinone, flavonoid, and tannin profiles [33,34,35,36,37].

The findings of the present study are consistent with this framework. Strong positive correlations were identified between flavonoid content and primary cortex thickness (*r* = 0.85), between tannin and flavonoid contents and xylem vessel area (*r* = 0.87 and *r* = 0.90, respectively), and between tannin content and periderm thickness (*r* = 0.88). These relationships suggest a coordinated functional linkage between anatomical traits and biochemical adaptations in response to altitudinal variation.

### 3.4. Role of Seasonal Climatic Factors

Statistical analysis revealed that both anatomical and phytochemical traits of *R. tataricum* ecopopulations were associated with seasonal variation in key climatic parameters across different growth localities. Periderm thickness exhibited positive correlations with winter precipitation and with relative air humidity in spring and summer (*r* = 0.91; *r* = 0.84; *r* = 0.86, respectively).

Organic acid content was identified as the metabolite most responsive to climatic variability, showing positive correlations with summer and autumn precipitation and a negative correlation with summer temperature (*r* = −0.85). In contrast, phenol, flavonoid, and tannin contents were positively correlated with winter precipitation, relative humidity, and autumn and winter temperatures.

These patterns are likely related to the ephemeroid life cycle of *R. tataricum*. During the short vegetative period (March–April), plants rely primarily on moisture accumulated during the cold season. Subsequently, the aboveground organs senesce, and seeds are dispersed by wind, occupying suitable micro-niches for germination in the following spring.

### 3.5. Soil Conditions as a Determinant of Phytochemical Profile

The soils within the habitats of the studied ecopopulations were characterised by low TOC content (data sourced from predictive model SoilGrids), a feature typical of desert-steppe and foothill ecosystems. Despite limited TOC availability, most wild *Rheum* species have been reported to occur preferentially on gravelly, carbonate, and slightly alkaline soils with relatively high calcium saturation [38]. Carbonate substrates have been associated with the development of a robust taproot system and dense rhizome tissues, facilitating access to deeper soil horizons and enhancing tolerance to drought and temperature stress [39].

Such edaphic conditions also influence the mineral composition of roots and rhizomes in the investigated ecopopulations. For instance, plants from the Sozaq district ecopopulation (Turkestan region), which develops on gypsum–carbonate desert soils characterised by high Ca content and elevated CEC (data sourced from predictive model SoilGrids), exhibited the highest Ca concentrations in root and rhizome tissues. A high Mg content in the roots, as observed in the Khantau population, may increase osmotic pressure in root tissues, thereby facilitating water uptake from saline solutions [40].

Soil texture, ranging from sandy to light loamy, determines the hydrological regime of the habitats and promotes conditions of periodic water deficit, which represents an important driver of ecological selection. Such conditions contribute to the shortened growing season and pronounced seasonality observed in rhubarb, enabling completion of active development during periods most favourable in terms of moisture availability. The time of the harvest period affects the BAC content [41]. Additional eustress factors such as the alkalinity and low salinity of soils observed may lead to the activation of water regulation and ionic balance mechanisms, thereby inducing stress-associated metabolic pathways involved in the accumulation of antioxidant compounds [19].

Alkaline carbonate substrates reduce the availability of Fe, Mn, and P, generating physiological stress and activating phenylpropanoid and polyketide pathways, which are central to AQ biosynthesis. Under such conditions, increased concentrations of emodin, aloe-emodin, and chrysophanol have been reported in rhubarb rhizomes [38]. Limited mobility of essential nutrients, particularly N and P, may further stimulate antioxidant defence systems and enhance the synthesis of secondary metabolites, including anthraquinones, flavonoids, and other phenolic compounds [38,42,43,44]. The present results are consistent with findings reported in the literature. However, it should be noted that the soil data used in this study were derived from a remote-sensing–based predictive model. Although studies evaluating the prediction accuracy and coverage probabilities of SoilGrids for the analysed parameters (0.89–0.93) support the scientific use of these predicted values and indicate a high level of data reliability [45,46,47], the need for further verification should be acknowledged. Specifically, the identified patterns and tendencies should be confirmed using analytically determined soil agrochemical profiles. Relatively high contents of AQs, phenols, and flavonoids were recorded in the Aq-Qainar population. In the Khantau ecopopulation, characterised by comparatively low soil TN availability, elevated concentrations of AQs, flavonoids, OAs, and tannins were also detected. The light-textured soils that facilitate periodic water deficit may further promote enhanced secondary metabolism, contributing to osmotic adjustment and protection of cellular structures against oxidative stress. Similar adaptive responses, including increased synthesis of BACs, have been described in other Polygonaceae species with metabolic profiles comparable to *Rheum* [44,48].

### 3.6. Integrative Interpretation and Significance of the Obtained Results

The combined influence of soil and climatic factors, including low humus content, carbonate–alkaline reaction, light soil texture, and periodic moisture deficit, appears to create a eustress-induced metabolic background that promotes enhanced synthesis of biologically active compounds in *Rheum tataricum*. These interacting drivers likely account for the observed spatial variability in chemical composition and for differences in the pharmacological potential of plant material collected under contrasting edaphic and climatic conditions. Altitudinal and latitudinal gradients, which shape the local soil and climatic environment, were found to exert a significant influence on both morphometric and phytochemical characteristics of the studied ecopopulations. The present findings support the concept that gradients in plant bioactive compounds rarely reflect the effect of a single environmental variable, but rather arise from the interaction of multiple stress factors [36]. The pronounced metabolic plasticity observed in *R. tataricum* ecopopulations is consistent with the capacity of a single genotype to express diverse anatomical, morphological, and chemical phenotypes under varying environmental conditions [20]. Further integrative investigations are warranted to elucidate and potentially manage the relationships among climate change, environmental stressors, and the biosynthesis of valuable plant-derived bioactive compounds [49].

## 4. Materials and Methods

### 4.1. Characterisation of Collection Sites

Roots and rhizomes of *R. tataricum* were collected from several ecopopulations located in different regions of Kazakhstan. The geographical coordinates of the sampling sites are presented in Table 4.

Climatic characteristics of the collection sites were compiled to describe the environmental conditions of each locality and are summarised in Table 5.

### 4.2. Climate Sensitivity Assessment in Collection Sites

To quantitatively evaluate the climatic vulnerability of *R. tataricum* ecopopulations, using monthly meteorological observations for the period 1991–2023, the Climatic Sensitivity Index (CSI) was calculated. The CSI is based on comparison of deviations in key biological parameters—yield, biologically active compound (BAC) content, flowering time, population density, and fertility—between years characterised by high climatic stress when extreme temperatures, precipitation and humidity lead to significant deterioration of plant health or destruction of the habitat. and the long-term climatic norm. The normative biological parameters calculated as an ICF index and correspond to years characterized by low climatic pressure (ICF < 0.3), while the anomalous values are composite long-term averages for seasons with increased climatic stress (ICF > 0.7).

The CSI was calculated according to the following equation [50]:

(1) $$CSI=\;\frac{1}{2}\sum_{i=1}^{N}\frac{X_{i\text{-}anomaly}-X_{i\text{-}norm}}{X_{i\text{-}norm}}$$

where *X_i_*—the *i*-th biological parameter (e.g., yield or BAS content); *X_i-norm_*—the mean value of the parameter in years with low climatic stress when temperatures, precipitation and humidity are within the optimum range for plant growth; *X_i-anomaly_*—the value recorded in years with high climatic stress; and *N*—the number of parameters included in the analysis (*N* = 5 in the present study).

The CSI is normalised to a range of 0–1 and was used to classify species according to their degree of climate sensitivity. The following thresholds were applied:

- CSI < 0.15—very low sensitivity (resistant species);
- 0.15 ≤ CSI < 0.30—moderately low sensitivity;
- 0.30 ≤ CSI < 0.50—high sensitivity;
- CSI ≥ 0.50—critical sensitivity (vulnerable species requiring conservation measures).

The ICF was calculated according to the following equation [50]:

(2) $$ICF=\;\frac{\omega_{1}(\frac{T_{min}+T_{max}}{2})}{T_{avg}}+\frac{\omega_{2}R}{R_{avg}}+\frac{\omega_{3}f}{f_{avg}}$$

where *T_min_* and *T_max_*—normalised minimum and maximum temperatures for a given month (°K); *R*—monthly precipitation; f represents the average relative humidity for the month; *T_avg_*—long-term average temperature for the growing season (°K); *R_avg_*—long-term average precipitation for the growing season; *f_avg_*—long-term average relative humidity for the growing season; *ω*_1_, *ω*_2_, and *ω*_3_—weighting coefficients for temperature, precipitation, and humidity, respectively, reflecting their relative contributions to vulnerability.

### 4.3. Soil Characteristics of Collection Sites

Soil parameters were obtained using the SoilGrids global digital soil model (ISRIC, Wageningen, The Netherlands; spatial resolution 250 m) for the 15–30 and 30–60 cm soil layers. The agrochemical characteristics of the sampling sites are summarised in Table 6.

### 4.4. Determination of Anatomical Traits

The anatomical structure of the collected samples was examined. Plant material (combined sample of ground roots and rhizomes from 5–7 plants per ecopopulation) was fixed in a mixture of ethanol, glycerol, and distilled water (1:1:1, *v*/*v*/*v*) prior to microscopic analysis.

Anatomical preparations were produced in accordance with established protocols in plant microtechnique [51,52,53], using a freezing microtome OL-ZSO (Inmedprom, Yaroslavl, Russia).

For quantitative analysis, morphometric traits were measured with an MOV-1-15 eyepiece micrometer (Lomo, Saint Petersburg, Russia) using a ×10 objective and magnifications of ×40, ×10, and ×7. Microphotographs of anatomical sections were obtained with an MC 300 microscope (Micros, Hunnenbrunn, Austria) equipped with a CAM V400/1.3M digital camera (jProbe, Yokohama, Japan).

Descriptions of macroscopic characters were prepared in accordance with the requirements of the State Pharmacopoeia of the USSR [54,55].

### 4.5. Determination of Mineral Composition

Samples of air-dried, ground plant material (3–5 g of combined sample of ground roots and rhizomes from 5–7 plants per ecopopulation) were subjected to dry ashing in porcelain crucibles to constant mass at 490–530 °C, followed by additional calcination at 600 °C until a uniform grey ash was obtained. The resulting ash was dissolved in dilute nitric acid (1:1, *v*/*v*), evaporated to near dryness to obtain moist salts, subsequently re-dissolved in 1 N hydrochloric acid (HCl) or nitric acid (HNO_3_), and diluted to a final volume of 25 mL.

Elemental composition was determined by atomic absorption spectroscopy using a DFS-13 spectrograph (wavelength range 2100–3600 Å; LOMO, St. Petersburg, Russia). Analytical accuracy was verified using certified reference material of copper sludge (Sh-MT CO 2962-84, 2964-84) [56].

### 4.6. Determination of Main Biologically Active Substances

#### 4.6.1. Quantitative Determination of Anthraquinones

Approximately 1 g of plant material (combined sample of ground roots and rhizomes from 5–7 plants per ecopopulation), ground to a particle size not exceeding 1 mm, was transferred into a 100 mL round-bottom flask fitted with a ground-glass joint. Subsequently, 15 mL of 10% sulphuric acid (H_2_SO_4_) was added, and the mixture was heated under reflux in a boiling water bath for 1 h. After cooling, 50 mL of chloroform was added, and the extraction was continued under reflux for a further 1 h.

The cooled extract was filtered into a separatory funnel. An alkaline ammonium solution (20 mL) was added, and the mixture was shaken for 5–7 min. Following complete phase separation, the transparent red lower layer was collected. The extraction procedure was repeated until the alkaline ammonium phase became colourless.

The optical density of the combined alkaline–ammonia extracts was measured at 525 nm using a LEKI SS2107UV spectrophotometer (MEDIORA OY, Helsinki, Finland) in a 10 mm path-length cuvette, with alkaline–ammonia solution used as the reference.

Anthraquinone (AQ) content was calculated according to the following formula:

(3) $$AQ=\;\frac{C\times 50\times 100\times 100}{m\times (100-W)}$$

where *C*—the concentration of anthraquinone derivatives in 1 mL of the test solution, determined from the calibration curve (g); *m*—the mass of the raw material sample (g); and *W*—the loss on drying of the raw material (%).

#### 4.6.2. Determination of Total Phenolic Compounds

Total phenolic content (TPC) was determined using the Folin–Ciocalteu colorimetric assay [57]. An ethanolic extract solution (1 mg mL^−1^) was used as the test solution. For analysis, 1 mL of extract (from combined sample of ground roots and rhizomes from 5–7 plants per ecopopulation) was mixed with 9 mL of distilled water (dH_2_O) in a 25 mL volumetric flask. Subsequently, 1 mL of Folin–Ciocalteu reagent was added, and the mixture was vigorously shaken. After 5 min, 10 mL of 7% (*w*/*v*) sodium carbonate solution was introduced, and the volume was adjusted to 25 mL with dH_2_O.

A series of gallic acid standard solutions (200–1000 μg mL^−1^) was prepared following the same procedure. After incubation for 90 min at room temperature, absorbance of both test and standard solutions was measured at 760 nm using a spectrophotometer, with dH_2_O serving as the control.

TPC was expressed as milligrams of gallic acid equivalents (GAE) per gram of extract (mg GAE g^−1^) [57]. All measurements were performed in triplicate.

#### 4.6.3. Determination of Organic Acid Content

An accurately weighed portion of plant material (10 g of combined sample of ground roots and rhizomes from 5–7 plants per ecopopulation), ground to a particle size not exceeding 2 mm, was placed in a flask, and 80 mL of purified water was added. The mixture was heated in a boiling water bath for 2 h. After cooling, the extract was filtered into a 100 mL volumetric flask, diluted to volume with dH_2_O, and thoroughly mixed.

An aliquot of 10 mL of the prepared solution was transferred into a 100 mL conical flask. An indicator mixture consisting of 1 mL of 1% ethanolic phenolphthalein solution and 2 mL of 0.1% methylene blue solution was added. The solution was titrated with 0.1 M sodium hydroxide (NaOH) until a stable purple-red colour appeared.

The percentage content of free organic acids (OAs) in absolutely dry plant material was calculated using the following formula:

(4) $$OA=\;\frac{V\times 0.0067\times 250\times 100\times 100}{m\times 10\times (100-W)}$$

where *V*—the volume of 0.1 M NaOH used for titration (mL); *m*—the mass of the raw material sample (g); and *W*—the loss on drying of the raw material (%). The coefficient 0.0067 corresponds to 0.0067 g of malic acid (or 0.01021 g of valerianic acid) equivalent to 1 mL of 0.1 M NaOH solution.

#### 4.6.4. Determination of Tannins

An accurately weighed portion of raw material (3 g of combined sample of ground roots and rhizomes from 5–7 plants per ecopopulation) was placed in a 100 mL conical flask, and 50 mL of hot purified water was added. The mixture was heated in a boiling water bath for 2 h. The aqueous extract was decanted, and the residue was re-extracted with an additional 50 mL of hot water under identical conditions. Combined extracts were transferred into a 100 mL volumetric flask and diluted to volume with purified water.

An aliquot of 10 mL of the prepared extract was transferred to a 500 mL conical flask. Subsequently, 100 mL of purified water and 10 mL of indigosulfonic acid solution were added, and the mixture was titrated with 0.02 M potassium permanganate (KMnO_4_) until a stable golden-yellow endpoint was achieved. A blank determination was performed by titrating 10 mL of indigosulfonic acid in 100 mL of purified water under identical conditions. One (1) mL of 0.02 M KMnO_4_ corresponds to 0.004157 g of hydrolysable tannins or 0.00582 g of condensed tannins, expressed as tannin equivalents [58,59].

Tannin content (TC), expressed as a percentage of absolutely dry raw material, was calculated using the following formula:

(5) $$TC=\;\frac{\left(V_{1}-V_{2}\right)\times K\times D\times V\times 100\times 100}{V_{3}-m\times (100-W)}$$

where *V*_1_—the volume of 0.02 M KMnO_4_ used for titration of the extract (mL); *V*_2_—the volume of 0.02 M KMnO_4_ used in the blank determination (mL); *V*_3_—the volume of extract taken for titration (mL); *V*—the total volume of the extract (mL); *m*—the mass of the raw material sample (g); *W*—the loss on drying of the raw material (%); *D*—the conversion factor corresponding to the type of tannins; and *K*—the correction factor.

#### 4.6.5. Determination of Flavonoid Content

Flavonoid content was determined spectrophotometrically using aluminium chloride (AlCl_3_) complex formation under acidic conditions, according to the method of Christ and Müller [60].

Briefly, 1 mL of plant extract (from combined sample of ground roots and rhizomes from 5–7 plants per ecopopulation) was mixed with 4 mL of dH_2_O in a 10 mL volumetric flask. Subsequently, 0.3 mL of 5% sodium nitrite (NaNO_2_) solution was added. After 5 min, 0.3 mL of 10% AlCl_3_ solution was introduced. Following a further 6 min incubation, 2 mL of 1 M NaOH was added, and the volume was adjusted to 10 mL with dH_2_O.

Absorbance of the test and standard solutions was measured at 430 nm using a LEKI SS2107UV spectrophotometer (MEDIORA OY, Helsinki, Finland), with the corresponding reagent mixture used as the control. Flavonoid concentration was calculated from a calibration curve prepared with quercetin standards (100–1000 μg mL^−1^). Total flavonoid content (TFC) was expressed as milligrams of quercetin equivalents (QE) per gram of extract (mg QE g^−1^). All measurements were performed in triplicate.

TFC, calculated as quercetin equivalents and expressed as a percentage of absolutely dry raw material, was determined using the following formula:

(6) $$TFC=\;\frac{D\times 100\times 100\times 25\times 100}{764.6\times m\times 2\times (100-W)}$$

where *D*—the optical density of the test solution; *m*—the mass of the raw material sample (g); 764.6—the specific absorption coefficient of the QE–AlCl_3_ complex at 430 nm; *W*—the loss on drying of the raw material (%).

### 4.7. Statistical Data Processing

Statistical analyses were performed using RStudio software (version 2023.06.0, build 421; RStudio PBC, Boston, MA, USA, 2023). One-way analysis of variance (ANOVA) was used to identify statistically significant differences among experimental variants. When significant effects were detected by ANOVA, Tukey’s honestly significant difference (HSD) test was applied for pairwise comparisons. Experimental variants were classified according to the test results using letter-based groupings in descending order at *p* < 0.05.

## 5. Conclusions

The present study demonstrated that *Rheum tataricum* L.f. exhibits pronounced anatomical and metabolomic plasticity, enabling the species to maintain viability under persistent climatic and edaphic stress in the arid regions of Kazakhstan. Altitudinal and seasonal climatic gradients were identified as key determinants of variability in rhizome anatomical traits and in the accumulation of biologically active compounds. Coordinated modification of anatomical structure and metabolomic profile contributes to the formation of an adaptive phenotype that supports effective protection, optimised water regulation, and enhanced biochemical resilience.

The findings have important practical implications for: (i) evidence-based bioprospecting of medicinal plant raw materials; (ii) the development of sustainable natural resource management strategies; (iii) conservation of the chemical diversity of wild *R. tataricum* populations; and (iv) the design of controlled cultivation systems aimed at producing plants with defined phytochemical profiles.

## Figures and Tables

**Table 1:** Climate Sensitivity Index values for the studied growing zones.

Species	Growth Region	Parameter	CSI	Sensitivity
*R. tataricum* L.f.	Southern Kazakhstan	Biomass productivity, g m^−2^	0.400	High
		BAC, % DM	0.314	High
		Florescence, day	0.333	High
		Average density, ind. m^−2^	0.385	High
		Generative shoots, %	0.300	High

Notes: CSI—climate sensitivity index; BAC—biologically active compounds; DM—dry matter.

**Table 2:** Morphometric traits of the anatomical structure of *R. tataricum* rhizomes from different localities. Different letters adjacent to the values indicate a significant difference between *R. tataricum* populations within the same morphometric trait.

Location	Periderm Thickness, µm	Primary Cortex Thickness, µm	Area of Xylem Vessels, ×10^−3^ mm^2^
Shoqysu	2.96 ± 0.25 **bc**	21.3 ± 0.55 **c**	0.36 ± 0.07
Sozaq	2.60 ± 0.23 **c**	22.1 ± 0.55 **c**	0.44 ± 0.09
Baqbaqty	3.60 ± 0.45 **bc**	24.0 ± 0.65 **b**	0.38 ± 0.07
Aqkol	3.31 ± 0.35 **bc**	26.6 ± 0.70 **a**	0.41 ± 0.08
Khantau	4.78 ± 0.48 **a**	26.6 ± 0.75 **a**	0.56 ± 0.09
Aq-Qainar	3.75 ± 0.43 **ab**	26.8 ± 0.70 **a**	0.49 ± 0.08
*p*-value	<0.001	<0.001	0.932

**Table 3:** Accumulation of EEs and PTEs in *R. tataricum* rhizomes collected from different sites. Different letters adjacent to the values indicate a significant difference between *R. tataricum* populations within one element at *p* < 0.05.

Element	Unit	Shoqysu	Sozaq	Baqbaqty	Aqkol	Khantau	Aq-Qainar
Essential elements (EEs)							
Na	g kg^−1^	3.30 ± 0.36 **ab**	4.00 ± 0.68 **a**	3.20 ± 0.48 **abc**	2.40 ± 0.22 **bc**	2.10 ± 0.13 **c**	2.80 ± 0.50 **bc**
Mg		3.60 ± 0.54 **ab**	4.30 ± 0.26 **ab**	3.20 ± 0.35 **b**	3.50 ± 0.63 **ab**	4.70 ± 0.42 **a**	4.00 ± 0.68 **ab**
K		7.40 ± 1.33 **c**	9.40 ± 0.85 **bc**	8.20 ± 0.57 **c**	20.1 ± 1.21 **a**	8.90 ± 1.34 **bc**	11.5 ± 1.27 **b**
Ca		34.6 ± 3.11 **b**	49.0 ± 7.35 **a**	30.2 ± 5.13 **bc**	18.4 ± 1.29 **cd**	38.8 ± 6.60 **ab**	16.5 ± 1.63 **d**
Potentially toxic elements (PTEs)							
Mn	mg kg^−1^	32.4 ± 3.56 **a**	27.0 ± 2.70 **ab**	12.8 ± 2.30 **d**	28.3 ± 3.11 **ab**	18.2 ± 1.09 **cd**	22.6 ± 1.58 **bc**
Fe		174 ± 15.7 **a**	164 ± 24.7 **a**	68.3 ± 4.10 **bc**	39.2 ± 7.06 **c**	95.9 ± 6.71 **b**	201 ± 18.1 **a**
Ni		5.20 ± 0.31 **b**	3.70 ± 0.33 **bc**	9.40 ± 1.13 **a**	3.30 ± 0.56 **c**	3.60 ± 0.43 **bc**	3.00 ± 0.30 **c**
Cu		4.50 ± 0.32 **a**	1.40 ± 0.15 **d**	3.20 ± 0.26 **b**	2.40 ± 0.43 **bc**	5.40 ± 0.43 **a**	2.00 ± 0.30 **cd**
Zn		6.80 ± 1.22 **c**	5.90 ± 0.35 **c**	12.0 ± 1.08 **c**	5.40 ± 0.81 **c**	71.5 ± 7.13 **a**	55.9 ± 5.03 **b**
Cd		0.80 ± 0.14 **ab**	0.70 ± 0.07 **ab**	0.90 ± 0.10 **a**	0.60 ± 0.04 **bc**	0.40 ± 0.04 **c**	0.70 ± 0.06 **ab**
Pb		6.90 ± 0.76 **b**	6.40 ± 0.38 **b**	12.0 ± 0.84 **a**	8.20 ± 0.81 **b**	3.90 ± 0.66 **c**	7.70 ± 0.46 **b**

**Table 4:** Geographical coordinates of *R. tataricum* collection sites.

Collection Site	Region—District—Village	Latitude, m a.s.l.	GPS Coordinates
Shoqysu	Aqtobe—Shalqar—vicinity of Shoqysu railway station	85	47°15′07.42″ N, 60°59′42.00″ E
Sozaq	Turkestan—Sozaq—54 km north of Sozaq	240	44°36′48″ N, 68°44′04″ E
Baqbaqty	Almaty—Balkhash—vicinity of Baqbaqty	414	44°44′76″ N, 76°71′91.53″ E
Aqkol	Zhambyl—Talas—northern shore of Lake Aqkol	441	43°42′80.73″ N, 70°65′72.43″ E
Khantau	Zhambyl—Moiynkum [Khantau Mountains]—20 km north-east of Khantau	558	44°23′59.9″ N, 73°52′36.6″ E
Aq-Qainar	Zhambyl—Turar Ryskulov—5 km north-east of Aq-Kainar	569	43°08′49.2″ N, 73°04′56.4″ E

**Table 5:** Climatic characteristics of the *R. tataricum* collection sites (average long-term data for 1991–2023).

Site	January	February	March	April	May	June	July	August	September	October	November	December
Mean monthly temperature, °C												
Shoqysu	−10.5	−9.4	0	11.8	19.7	25.8	27.7	25.7	18.1	9.2	−0.6	−7.9
Sozaq	−5.5	−3.3	4.1	12.2	18.9	24.9	26.8	24.9	18.1	10.1	1.8	−4.1
Baqbaqty	−8.0	−6.0	1.2	9.6	15.0	20.0	22.0	21.1	15.0	7.8	0.6	−5.9
Aqkol	−6.3	−3.8	4.6	13.1	19.6	25.4	27.2	25.3	18.4	10.4	1.8	−4.5
Khantau	−6.5	−3.3	5.2	13.2	18.7	23.8	25.6	23.9	18.0	10.4	2.1	−4.6
Aq-Qainar	−4.7	−2.3	4.9	11.9	17.3	22.7	25.1	23.5	17.5	10.2	2.7	−3.3
Relative humidity, %												
Shoqysu	84.0	82.0	76.0	53.0	45.0	37.0	37.0	37.0	43.0	58.0	76.0	82.0
Sozaq	79.0	77.0	70.0	56.0	47.0	34.0	33.0	32.0	37.0	53.0	72.0	78.0
Baqbaqty	77.0	77.0	74.0	61.0	56.0	49.0	48.0	45.0	46.0	61.0	73.0	77.0
Aqkol	78.0	73.0	67.0	55.0	48.0	37.0	35.0	35.0	41.0	54.0	70.0	77.0
Khantau	84.0	81.0	72.0	59.0	56.0	49.0	47.0	46.0	50.0	64.0	78.0	84.0
Aq-Qainar	81.0	80.0	76.0	67.0	60.0	48.0	42.0	41.0	46.0	63.0	76.0	82.0
Mean monthly precipitation, mm												
Shoqysu	11.0	11.0	16.0	14.0	13.0	12.0	8.0	6.0	4.0	11.0	14.0	13.0
Sozaq	14.0	16.0	25.0	30.0	25.0	15.0	8.0	2.0	3.0	13.0	18.0	16.0
Baqbaqty	21.0	22.0	31.0	35.0	43.0	42.0	41.0	20.0	14.0	34.0	33.0	23.0
Aqkol	16.0	17.0	19.0	23.0	15.0	8.0	9.0	3.0	4.0	13.0	20.0	17.0
Khantau	27.0	29.0	26.0	38.0	32.0	24.0	16.0	11.0	10.0	30.0	35.0	32.0
Aq-Qainar	22.0	26.0	35.0	51.0	43.0	23.0	15.0	7.0	12.0	30.0	37.0	27.0

**Table 6:** Agrochemical parameters of soils at the collection sites (data derived from SoilGrids).

Parameter	Units	Soil Horizons, cm	Shoqysu	Sozaq	Baqbaqty	Aqkol	Khantau	Aq-Qainar
pH (H_2_O)	−	15–30	8.20	7.90	7.80	7.70	7.90	7.70
		30–60	8.10	7.80	7.90	7.90	8.10	8.10
CEC	cmol(c) kg^−1^	15–30	25.8	35.4	23.2	23.2	17.9	21.5
		30–60	25.9	34.8	22.5	24.5	17.6	21.6
SOC	g kg^−1^	15–30	22.4	12.1	22.8	6.70	8.80	9.40
		30–60	22.9	9.70	24.8	4.20	5.50	6.90
TN	g kg^−1^	15–30	1.46	1.23	1.55	0.95	0.84	1.35
		30–60	1.37	0.79	1.10	0.89	0.64	0.75

Notes: CEC—cation exchange capacity; SOC—soil organic carbon; TN—total nitrogen.

## Data Availability

The original contributions presented in this study are included in the article. Further inquiries can be directed to the corresponding authors.

## Abbreviations

The following abbreviations are used in this manuscript:

AQ(s)	anthraquinone(s)
Au	autumn
BAC(s)	biologically active compound(s)
CEC	cation exchange capacity
CSI	climate sensitivity index
DM	dry matter
EE(s)	essential for plant growth element(s)
m a.s.l.	metres above sea level
OA(s)	organic acid(s)
PTE(s)	potentially toxic element(s)
*R. tataricum*	*Rheum tataricum* L.f.
SOC	soil organic carbon
Sp	spring
Su	summer
TN	total nitrogen
Wi	winter
