# Correction: Terletskaya et al. Soil–Climatic Drivers of Anatomical and Metabolic Plasticity in *Rheum tataricum* L.f. Across Arid Landscapes of Kazakhstan. *Plants* 2026, *15*, 1025

**DOI:** 10.3390/plants15121767

**Published:** 2026-06-08

**Authors:** Nina Terletskaya, Aigerim Mamirova, Yuliya Litvinenko, Meruyert Kurmanbayeva, Svetlana Polyakova, Nadezhda Gemejiyeva, Timur Kulmanov, Vitaliy Salnikov, Aizhan Mussayeva

**Affiliations:** 1 Faculty of Biology and Biotechnology, Al-Farabi Kazakh National University, Al-Farabi 71/19, Almaty 050040, Kazakhstan; aigerim.mamirova@mail.com (A.M.); meruyert.kurmanbayeva@kaznu.kz (M.K.); 2 Institute of Genetic and Physiology, Al-Farabi 93, Almaty 050040, Kazakhstan; rumex1978@gmail.com (Y.L.); aimus_@mail.ru (A.M.); 3 Faculty of Chemistry and Chemical Technology, Al-Farabi Kazakh National University, Al-Farabi 71/19, Almaty 050040, Kazakhstan; 4 Faculty of Geography and Environmental Sciences, Al-Farabi Kazakh National University, Al-Farabi 71/19, Almaty 050040, Kazakhstan; polse2468@gmail.com (S.P.); vitali.salnikov@kaznu.edu.kz (V.S.); 5 Institute of Botany and Phytointroduction, Almaty 050040, Kazakhstan; ngemed58@mail.ru

Additional Affiliation

In the published publication [1], there was an error regarding the affiliations for Aigerim Mamirova. In addition to affiliation 1, the updated affiliations should include 1,2. 

In the published publication [1], there was an error regarding the affiliations for Yuliya Litvinenko. In addition to affiliation 3, the updated affiliations should include 2,3. 

Correction of the Name and Address of Affiliation No. 4:

During the proofreading process, the previous faculty name was retained. The affiliation should be updated to reflect the current official institutional name. The requested correction is as follows: Faculty of Geography and Environmental Sciences, Al-Farabi Kazakh National University, Al-Farabi 71/19, Almaty 050040, Kazakhstan.

Addition of an Author

Vitaliy Salnikov was not included as an author in the original publication. The corrected Author Contributions statement appears here. 

Conceptualization, N.T.; methodology, M.K., Y.L., S.P. and V.S.; software, A.M. (Aigerim Mamirova); formal analysis, T.K.; investigation, Y.L., S.P., N.G. and M.K.; resources, A.M. (Aizhan Mussayeva); data curation, N.T.; writing—original draft preparation, M.K., Y.L. and S.P.; writing—review and editing, N.T. and A.M. (Aigerim Mamirova); visualization, A.M. (Aigerim Mamirova); supervision, N.G.; project administration, A.M. (Aizhan Mussayeva); funding acquisition, N.T. All authors have read and agreed to the published version of the manuscript.

The authors state that the scientific conclusions are unaffected. This correction was approved by the Academic Editor. The original publication has also been updated.
